# *In Vivo* Anthelmintic Efficacy of *Adansonia digitata* and *Anogeissus leiocarpa* against *Haemonchus contortus* Infestation Induced in Sheep

**DOI:** 10.1155/2024/3249640

**Published:** 2024-08-30

**Authors:** Julienne Kuiseu, Basile Konmy, Christian Cocou Dansou, Tony Taofick Babalola Abiodoun Sounkere, Claude Gbemeho Houssoukpe, Sylvie Mawule Hounzangbe-Adote, Patrick Aleodjrodo Edorh, Pascal Abiodoun Olounlade

**Affiliations:** ^1^ Zootechnics and Livestock Systems Research Unit (URZoSE) National University of Agriculture (UNA), Kétou, Benin; ^2^ Laboratory of Toxicology and Environmental Health (LATSE) University of Abomey-Calavi (UAC), Abomey-Calavi, Benin; ^3^ Laboratory of Ethnopharmacology and Animal Health (LESA) University of Abomey-Calavi (UAC), Abomey-Calavi, Benin

## Abstract

The aim of the present study was to assay the *in vivo* anthelminthic activity of *Anogeissus leiocarpa* (Al) family of the Asteraceae and *Adansonia digitata* (Ad) family of Malvaceae leaf powder against the nematode *Haemonchus contortus* (Hc) in sheep. Twenty-eight sheep were artificially infected with 3000 Infective larvae (L3) of Hc and divided into four groups. Groups 1 and 2 received 3.2 g/kg of Ad and Al leaf powder according to the body weight for three days by oral route. This treatment was repeated after 14 days. Group 3 received albendazol 5 mg/kg and group 4 received water. The treatment was repeated 14 days later. Examination of faecal samples, packed cell volume and biochemical analyses and necropsy were carried out to determine egg counts, worm burdens, and reduction in worm fecundity and changes in blood parameters. The results showed a reduction in egg excretion of 72.22% and 88.49%, respectively, with Al and Ad leaf powder. Egg laying of adult female worms was reduced by 55.22% and 64.96% with Ad and Al, respectively. FAMACHA score (≤2 in the treated animals) and packed cell volume were improved with Ad and Al. The results of this study revealed that Ad and Al powder may be used as an alternative anthelminthic to control haemonchosis in small ruminants.

## 1. Introduction

Small ruminants arouse great interest in Benin. These are animals that are involved in programs to improve the level of national animal production given their low food requirements, their short reproduction cycle compared to large ruminants, and their rustic character, allowing them to evolve in the conditions of most difficult breeding [1] and increase the income of breeders [2]. Nevertheless, the breeding of small ruminants is characterized by low zootechnical productivity and insufficient health supervision [3]. This is explained by the method of farming, mainly focused on the exploitation of pastures, which promotes gastrointestinal parasitism, one of the major limiting factors of this activity in the tropics [3]. Indeed, it appears from the parasitological surveys carried out in small ruminants in Benin by Salifou [4], that the parasitic fauna of their digestive tract is dominated by strongyles mainly *Haemonchus contortus*, *Trichostrongylus colubriformis*, and *Oesophagostomum columbianum*. The overall prevalence of Hc found in Benin in sheep was estimated at 92.5% [5]. Moreover, Hc parasite is a hematophage, responsible for significant production losses in sheep and goat farms [6]. It causes an alteration of the general condition of animals through digestive disorders, weight loss, anemia, alteration of wool quality and reproductive capacity [6]. For decades, the control of infestations due to gastrointestinal nematodes has relied essentially on the repeated use of synthetic anthelmintics [7]. The uncontrolled use of these drugs has led to the development of resistance in gastrointestinal nematodes, with the corollary of considerable economic losses to farmers. [8]. An alternative to these problems, explored by farmers in sub-Saharan African communities, is the use of ethno-veterinary medicine [9], through the use of medicinal plants with anthelmintic properties to combat animal parasitic diseases [10]. This is justified by the availability, accessibility, and affordable cost of medicinal plants vis-à-vis local populations, especially those with low incomes [11].

Through the studies carried out previously, it is observed that in developing countries, more than 80% of the population use medicinal plants in first intention because of their easy access compared to modern drugs [12]. Thus, bioactive plants with anthelmintic properties today constitute one of the main alternative or complementary solutions to the anthelmintics explored [7]. Among these plants are Ad (Baobab) and Al (African Birch) known for their multiple properties. A bibliographical synthesis of the two plants revealed that they possess anthelmintic, analgesic, and antimicrobial properties [13–18]. Studies conducted on African flora medicinal plants have reported that different parts of Al, mainly the leaves, are used to control and treat gastrointestinal parasitism of small ruminants by farmers in West Africa, especially in Burkina-Faso [19, 20]; in Nigeria [21, 22]. The barks are also used by small farmers to control helminth infections in small ruminants in Nigeria [23], in Burkina Faso [19]. In Côte d'Ivoire, these plants are used to control helminths [24]. The leaves, bark, and fruit pulp of Ad are traditionally used as an immunostimulant, anti-inflammatory, analgesic, insect repellent, pesticide, and as a treatment for worms [25]. Ad and Al leaves are very rich in polyphenolic compounds such as tannins alkaloid, quinone, and flavonoids [26–30]. The presence of these secondary metabolites may explain the use of these two plants in the treatment of gastrointestinal worms in humans and animals.

The present study aims to evaluate the anthelmintic activity of *Adansonia digitata* and *Anogeissus leiocarpa* leaf powder against *Haemonchus contortus*.

## 2. Materials and Methods

### 2.1. Study Framework

The animal experiments were carried out at the Zootechnics and Livestock Systems Research Unit (URZoSE) of the Laboratory of Animal and Fisheries Sciences (LaSaH) of National University of Agriculture (UNA) of Benin.

### 2.2. Harvesting and Preparation of Plants

*Adansonia digitata* and *Anogeissus leiocarpa* leaves had been harvested in the commune of Kétou in southern Benin. The leaves were harvested from mature plants with healthy and robust foliage. The harvest was done during the rainy season when the plant growth is vigorous. Knives were used to harvest the leaves. The healthiest and most vigorous leaves of the plant were selected, avoiding harvesting all the leaves from a single branch or plant. The leaves of the both plants were authenticated at the National Herbarium of University of Abomey-Calavi under the numbers YH 481/HNB and YH 482/HNB, respectively. The leaves were sorted, and those that were eaten by insects or deformed were discarded. The selected leaves were carefully cleaned with water to remove dirt, dust, or insects. The cleaned leaves were spread out in the laboratory and dried at 20°C for 14 days. The dry leaves were ground using an electric grinder. The powders obtained were stored in airtight bottles.

### 2.3. Animal and Housing

Twenty-eight Djallonké sheep of 4 months old, with an average weight of 8.9 ± 1.41 kg were used for this study. The animals were housed in individual, well-ventilated pens and identified by numbered wooden tags. They received prophylactic care and were given 20% oxytetracycline for 3 days. These animals received albendazole and ivermectin at the recommended dose.

### 2.4. Experimental Design

**Figure 1:**
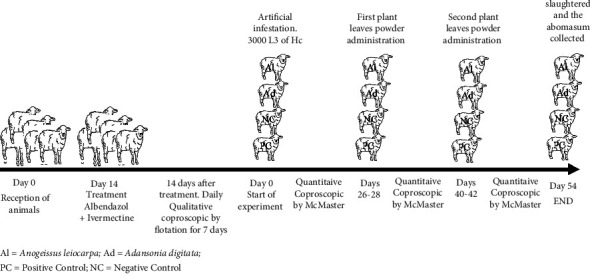
Experimental design.

After 21 days of quarantine, 28 sheep were divided into 4 homogenous groups of 7 sheep. Qualitative faecal examinations were conducted every day, 14 days after quarantine to ensure the total absence of parasites in the animals before proceeding with artificial infestation. The sheep received 3000 L3 of Hc larvae by oral gavage according previous study [31]. The animals were housed individually in cages. They were fed concentrates and a supplement of cassava peels *ad libitum*. They had access to drinking water and a salt lick. 21 days postinfestation; groups 1 and 2 animals received 3.2 g/kg of body weight of Al and Ad leaf powder by oral gavage during 3 days according previous study [32]. Group 3; positive control received albendazole 5 mg/kg and group 4; negative control received water. Treatments with Al and Ad leaf powder were repeated 14 days later. The animals were fed a commercial concentrate and *Manihot esculenta* peelings during the trial (Figure 1).

### 2.5. Indirect Evaluation of the Effects of Anthelmintic Plants

#### 2.5.1. Quantitative Coproscopic

The number of eggs present in the faeces taken directly from the rectum was analyzed on the same day in the laboratory by the method of McMaster [33]. Its usefulness for assessing the degree of parasite infestation is limited by factors modulating egg shedding [34]. Parasite reduction rates relative to the control were calculated using the following formula:

(1) $$\begin{array}{c} \text{RR}=\frac{\text{EPG before Treatment}-\text{EPG after Treatment}}{\text{EPG before Treatment}}\times 100. \end{array}$$

With RR = Reduction Rate

#### 2.5.2. Determination of the Packed Cell Volume

Blood was collected via puncture of the jugular vein and placed in tubes containing EDTA as an anticoagulant for packed cell volume (PCV) assessment using microcentrifugation, following the microhematocrit method described in previous study [32].

#### 2.5.3. Determination of FAMACHA Scale's Variation

The FAMACHA system is based on the observation of the pallor of the mucous membranes in infected animals. This system is based on a semiquantitative evaluation of the color of the mucous membranes of the eyes, which is categorized from 1 (red, not anemic) to 5 (white, severely anemic) [35]. Animals were weighed weekly and carried out using a 20 ± 0.2 kg load cell. It made it possible to follow the weight evolution of the animals during the test.

#### 2.5.4. Blood Sampling

Blood samples were obtained from the jugular vein by a certified veterinarian, as described by [36]. Two types of tubes were used: one without additives for biochemical analysis, and another containing EDTA as an anticoagulant for hematological analysis. After collection, the tubes were centrifuged at 3000 g for 10 minutes to separate the serum for biochemical analysis. Hematological analysis was conducted using whole blood samples, following the protocol outlined by [37].

#### 2.5.5. Biochemical and Hematological Analyses

Biochemical analyses included the determination of alkaline phosphatase, gamma-glutamyl transferase, total bilirubin, and conjugated bilirubin using spectrophotometry methods. Hematological parameters such as white blood cell count (WBC), red blood cell count (RBC), hemoglobin (Hb), packed cell volume (PVC), mean corpuscular volume (MCV), mean corpuscular hemoglobin (MCH), and mean corpuscular hemoglobin concentration (MCHC) were determined according to the procedures described in previous studies [37].

### 2.6. Direct Parasitic Assessment

At the end of the experiment (days 54 postinfestation), the animals were slaughtered and the abomasum collected. The parasite assessment technique consists of recovering organs from the digestive tract of sacrificed animals just after slaughter and analyzing their contents. This technique is the only one that provides a reliable estimate of the real parasite load of the animals and is considered the reference method as opposed to coproscopic which is considered much more random.

#### 2.6.1. Collection of Worms in the Abomasum

The contents of the abomasum are recovered and rinsed with lukewarm saline (37°C) in order to collect all the parasites, present, both immature worms and adult worms. The abomasum collected from the sacrificed animal was washed on the internal face, turned towards the bottom of the container, and left to soak for approximately one hour. The organs were then removed and carefully rinsed with physiological saline. The soaking and second rinsing solutions are added to the first rinsing liquid and the volume adjusted to 500 mL or one liter. To this mixture is added 70% ethanol and formalin for conservation and for subsequent use.

#### 2.6.2. Worm Count

The observations were made with a binocular magnifying glass and the identification of the parasites was made according to several criteria which are: the size, morphology of the anterior end, and the appearance of the caudal bursa in males. Adult and immature worms were counted per aliquot part (AP) of 10% of the total volume. If the number of worms is less than or equal to 10, then the worms are counted in 20% of the volume and the result is extrapolated to the initial volume by a rule of three.

#### 2.6.3. Determining the Fecundity of Worms

This technique described by Kloosterman et al. [38] determines the individual fertility of Hc females. The worms collected during the assessments were kept in a 70° ethanol solution. They are put in water for rehydration for 5 minutes. The female worms are introduced individually into 200 *μ*l of hypochlorite solution diluted to 1/5 and left for 2 to 5 min for them to burst. Counting of released eggs was done in 30% of the total volume using a binocular magnifying glass. The number (N) obtained is related to the total according to the formula:

(2) $$\begin{array}{c} N=\frac{N1+N2}{30}\times 100, \end{array}$$

*N*1 and *N*2 = number of eggs counted in the 2 aliquots.

### 2.7. Ethical Considerations

The experimental guideline and animal welfare were conformed to the animal research guideline adopted by the Doctoral School of Agronomic and Water Sciences (EDSAE) ethics committees of National University of Agriculture, Porto-Novo, Benin.

### 2.8. Statistical Analyses

The comparison of faecal excretion in the two groups of animals was carried out after log transformation (*x* + 1) of the coproscopy values. Comparisons of packed cell volume and fecundity per female of Hc present were made by means of a nonparametric test of comparison of means (ANOVA test) with R 4.2.1. software.

## 3. Results

### 3.1. Effect of Treatment on Faecal Excretion of Hc Eggs in Animals

**Figure 2:**
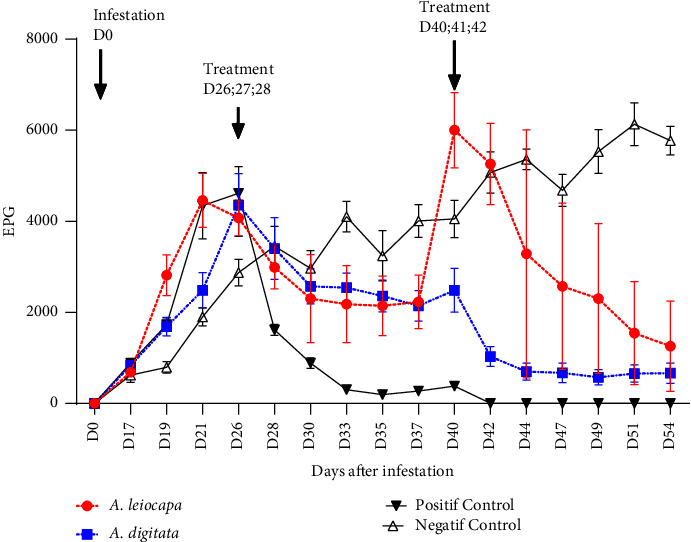
Variation in the rate of faecal excretion.

The rate of parasitism was 3978.57 ± 385.34 EPG on the day of the first treatment (day 22 postinfection). This explains the success of the infestation. This rate was considerably reduced to 1257.14 ± 992.79 EPG; 664.28 ± 23.38 EPG and 6128.571 ± 467.24EPG, respectively, in sheep treated with Al; Ad (day 54 postinfection) and negative control. The percentage efficacy calculated at day 54 postinfection was 72.22% and 88.49%, respectively, in sheep treated with Ad and Al. An efficacy of 100% was recorded in the positive control sheep. However, the variation in EPG in the negative control remained increasing throughout the experiment (Figure 2).

### 3.2. Effect of Treatment on Packed Cell Volume

**Figure 3:**
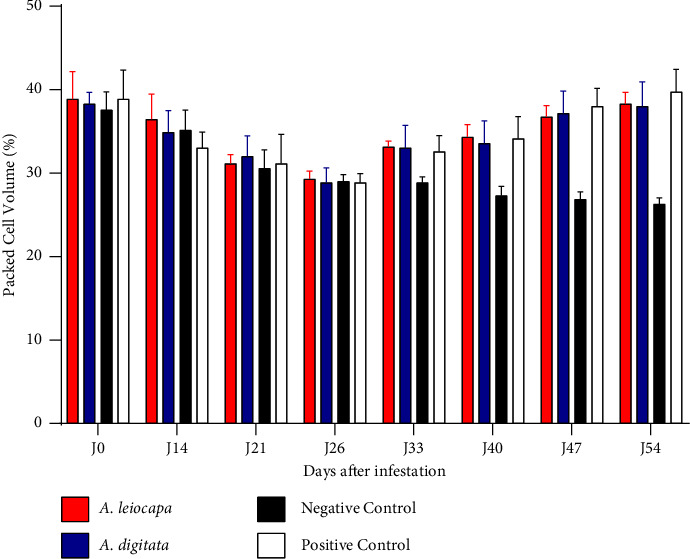
Variation of packed call volume of sheep.

The average PCV rate was 38.39 ± 0.31% at the start of the experiment. This level decreased overall to reach a value of ±0.1% on the first day of infestation. The PCV levels were improved in treated sheep compared with the negative control (Figure 3). Nevertheless animals of the negative controls were not considered anemic (<24 PCV).

### 3.3. Effect of Treatment on FAMACHA Scale Variation

**Figure 4:**
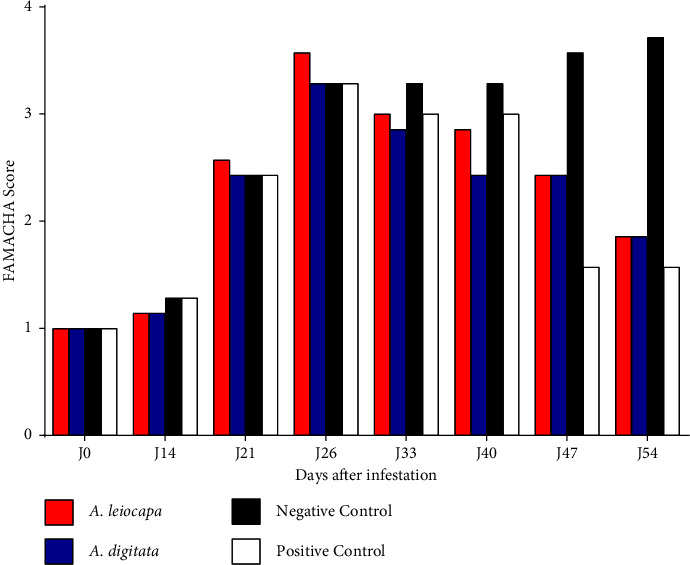
Variation of FAMACHA score of sheep.

At the start of the study, all animals had a FAMACHA score = 1. On the first day of treatment after infestation (D26), the animals had a FAMACHA score ≥3. During the treatment, the FAMACHA score improved to reach a FAMACHA score ≤2 in the treated animals; in contrast to the FAMACHA score ≥3 in the negative control (Figure 4).

### 3.4. Effect of Treatment on the Viability of Adult Worms

The administration of Al and Ad leaf powder significantly reduced (*p* < 0.01) the population of Hc acute worms present in the abomasum of sheep after postmortem examination at the day 54 postinfection. The reduction rate of worms is estimated at 58.61%, 62.79%, and 100%, respectively, for Ad; Al and Albendazole compared to the negative control (Table 1).

### 3.5. Effect of Treatment on the Fertility of Female Worms

At the end of the experiment, the leaf powder of the two plants was significantly reduced (*p* < 0.001) and the fecundity of the female worms expressed as the number of eggs counted after the uterus of each female worm burst (Table 2).

### 3.6. Effects of Al and Ad Powder on Some Hematological Parameters in Sheep after Infestation

Red blood cells and monocytes showed no significant difference (*p* > 0.05). Hemoglobin and packed cell volume (PCV) levels increased significantly (*p* < 0.05) compared to the positive and negative control. The globular constants VGM, CCMH, and TCMH significantly decreased (*p* < 0.01) compared to controls (Table 3).

### 3.7. Effects of Al and Ad Powder on Some Biochemical Parameters in Sheep after Infestation

**Figure 5:**
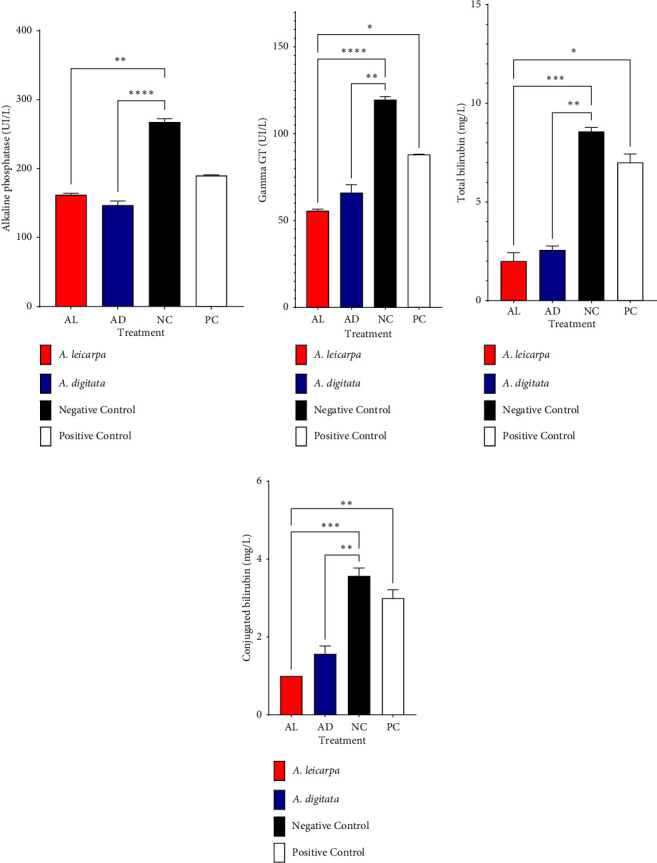
Variation of biochemical parameters in sheep after treatment. (a) Alkaline phosphatase; (b) gamma glutamine transferase; (c) total bilirubin; (d) conjugated bilirubin. Al: *A. leiocarpa*; Ad: *A. digitata*; NC: negative control; PC: positive control; Tukey test for independent samples. Dunn's test for multiple comparisons. ns: not significant; ^∗^ = *p* < 0.05; ^∗∗^ = *p* < 0.01; ^∗∗∗^ = *p* < 0.001.

The negative control presented the highest levels of alkaline phosphatase, gamma GT, and total and conjugated bilirubin (*p* < 0.05), on the other hand, the animals having received the powders of Ad and Al presented lower levels of alkaline phosphatase, gamma GT, and total and conjugated bilirubin (Figures 5(a) and 5(d)).

## 4. Discussion

This study assessed the *in vivo* efficacy of Ad and Al leaf against Hc egg excretion in artificially infected sheep. Indeed, Ad and Al leaves meals used in the present study had an anthelmintic effect on Hc egg excretion. The reduction rate of Hc egg excretion by the both plant is higher than the results of Ibrahimet al. [23], having obtained a deworming rate of 60% in the rat *Nippostrongylus braziliensis* with the methanolic extract of the bark of Al at a dose of 20 g/kg. *S. mombin* (Anacardiaceae) leaf powder reduces the rate of Hc egg excretion by 60.41% in goats (Akouedegni et al. 2019). According to Santos et al. [39], the ruminal flora is one of the factors that can have a strong influence on the activity of substances administered orally, as were the leaf powders of Al and Ad. After the second treatment, the condensed tannins and flavonoids level are likely to diffuse into the blood to induce many mechanisms (egg hatching, envelopment, and migration of L3 larvae, and inhibition of worm motility), thus causing further egg reduction.

The work carried out by Dramane et al. [40] showed that the ethanolic extract of the roots of Al at a single oral dose of 80 mg/kg revealed the anthelmintic effect of the plant in sheep through a moderate reduction in faecal eggs (81%), a reduction in adult worm load of Hc (87.4%), *Trichostrongylus coubriformis* (81.7%), high efficacy against adult *Strongyloides papillosus* (100%), *Gaigeria pachyscelis* (90%), *Cooperia curticei* (100%), and *Oesophagostomum columbianum* (95%) but low efficacy against *Trichostrongylus axei* (67%) and *Trichuris globulosa* (79%). This shows the effectiveness of the plant in controlling gastrointestinal parasitism. The results obtained by Dramaneet al. [40] on faecal egg reduction are similar to the results of the present study (88% with Al powder). However, the worm load of Hc adults obtained by Dramaneet al. [40] differs and is clearly superior to that obtained in the present study (62.79% with Al powder and 58.61% with Ad powder). This difference could be explained by the organs used (roots on the one hand, and leaves on the other), the type of plant material used for the *in vivo* experimentation (ethanolic extract on the one hand, and powder of somewhere else). However, Agaieet al. [21] revealed with the aqueous leaf extract of Al reduction rates in faecal excretion ranging from 15.2% to 20.5%; respectively, obtained with treatments of 200 and 400 mg/kg while the consecutive administration of 400 mg/kg for 3 days produced a reduction of 39.5%. Kaboré [3] indicates a level of reduction in egg excretion varying from −0.02% (D7) to 32.6% (D14) in the groups treated at different concentrations (250, 500, and 1000 mg/kg) with the aqueous leaf extract of Al. The results obtained by these authors on the faecal excretion of eggs differ and are clearly lower than the results obtained in the present study (88% and 66%, respectively, for the powders of Al and Ad). This could be explained by the difference in dose, the breed of sheep, or the type of plant material used during the various experiments (leaf powder in the present study and aqueous leaf extract in theirs). Plant-induced decreases in adult worms and female worm fecundity may be associated with the capacity of their bioactive compounds, including condensed tannins. In addition, Martínez-Ortíz-de-Montellano et al. [41] studied using an electron microscope. In previous studies of Kuiseuet al. [26], the phytochemical study of AL and Ad revealed the presence of numerous secondary metabolites such as tannin and flavonoids. These metabolites can easily explain the effect of these two plants on the reduction of faecal Hc eggs [26].

Otherwise, Agaieet al. [21] claim to have observed no significant change (*p* > 0.05) in the body weight of the groups treated with albendazole or the extract [21]. It is the same with [3] who, having exploited the natural pasture, did not observe any significant difference between the control batch and the groups treated with albendazole and the aqueous leaf extract of Al in Mossi breed sheep (*p* > 0.05). These results differ from those obtained by Dramaneet al. [40] (showing an increase in body weight from 0.7 ± 2.9 to 3.3 ± 1.9%), as well as from the findings of the present study, where the live weight of the animals was significantly improved (*p* < 0.05) in the treated groups (plant powders and albendazole) and in the neutral control. The difference may be justified by the type of plant material used for the experiment, the breed of sheep, and the farming method undertaken.

The anthelmintic effect of Al and Ad was due to the presence of secondary metabolites such as tannins, flavonoids, and polyphenols present in the leaves of these two plants [26]. Tannins, flavonoids, and essential oils are the secondary metabolites responsible for the anthelmintic activity of medicinal plants with anthelmintic potential [42, 43]. However, certain other compounds such as terpenoid and steroid compounds, anthracenosides, and saponosides [44]; alkaloids, flavonoids, saponosides, tannins, glycosides, reducing compounds, and anthraquinones [10, 45, 46] are also responsible for the anthelmintic properties of plants. Recent studies show that other secondary compounds such as hydroxycinnamic acids and phenolic acids (gallic acid) are associated with the anthelmintic effect [47–50].

Other studies have shown that aqueous and hydroethanolic extracts of *A. digitata* and *A. leiocarpus* exert effective anthelmintic activity on the inhibition of egg hatching and on the motility of *H. contortus adults* [51]. *Anethum graveolens* essential oil showed activity in inhibiting egg hatching, inhibiting larval development, and inhibiting migration of *H. contortus* larvae [52].

Agaieet al. [21] indicate that the aqueous leaf extract of Al had no significant effect (*p* > 0.05) on vital parameters such as temperature respiration and pulse rate. This result is similar to that obtained in the present study.

Packed cell volume improved from 0.7 ± 2.9 (week 1) to 3.3 ± 1.9% (week 3) after treatment [40]. This agrees with the result obtained in the present study because the packed cell volume levels, having decreased (*p* < 0.01) after the infestation from D0 to D26, increased significantly following the two treatments. These results are contrary to the results obtained by Kaboré [3].

## 5. Conclusion

In conclusion, the leaf powder of Al and Ad at a dose of 3.2 g/kg PV showed significant efficacy on faecal excretion, adult worm viability, and female worm fecundity in sheep, regardless of breed, when applied by a 3-day administration which was repeated 2 weeks later. Therefore, herbal preparation has proven to be an alternative way to replace or supplement the use of chemical drugs to achieve longer-lasting control of haemonchosis in West African sheep. Nevertheless, further *in vivo* studies are needed to assess the efficacy of these plants at the same dose against other gastrointestinal nematodes prevalent in small ruminants in tropical regions.

## Figures and Tables

**Table 1:** Variation in the number of adult worms in the abomasum treatment.

Treatments	Dose (g/kg)	Male	Female	Total
Ad	3.2	656.67 ± 50.64^b^	656.67 ± 43.102^b^	1313.33 ± 206.17^b^
Al	3.2	637.5 ± 42.735^b^	543.33 ± 28.944^c^	1180.83 ± 131.01^b^
Albendazole	5 × 10^−3^	0 ± 0^a^	0 ± 0^a^	0.00 ± 0^a^
Negative control	—	1340 ± 157.565^a^	1833.33 ± 353.786^d^	3173.33 ± 1124.75^c^
Test statistics	—	19.661	20.69	19.87
pvalue	—	0.001	0.001	0.001

The values a and b represent the means ± SEM, *n* = 6. The letters compare in the same column the results of the various treatments with respect to the control. Significant difference at *p* < 0.05.

**Table 2:** Variation in the number of eggs per female worm treatment.

Treatments	Dose (g/kg)	Eggs per female worm	Reduction rate (%)	*p* value
Ad	3.2	261.57 ± 50.91^b^	55.22	<0.001
Al	3.2	204.67 ± 76.33^b^	64.96	
Negative control	5 × 10^−3^	584.11 ± 50.21^a^		

The values a and b represent the means ± SEM. The letters compare in the same column the results of the various treatments with respect to the control. Significant difference at *p* < 0.05.

**Table 3:** Variations of some hematological parameters in sheep treatment.

Parameters	Al	Ad	NC	PC	*p* value
Hb	14.50 ± 0.28^ab^	14.66 ± 0.68^ab^	10.86 ± 0.09^b^	13.11 ± 0.08^a^	0.035
RBC	5.09 ± 0.10	5.19 ± 0.24	4.24 ± 0.03	4.72 ± 0.18	0.204
PVC	43.29 ± 0.81^ab^	43 ± 1.31^b^	31.57 ± 0.20^b^	39.57 ± 0.20^a^	0.004
VGM	85.08 ± 0.03^a^	84.93 ± 0.08^a^	85.49 ± 0.17^b^	84.89 ± 0.11^a^	0.001
MCHC	33.42 ± 0.10^ab^	33.61 ± 0.02^ab^	34.09 ± 0.24^b^	33.29 ± 0.07^a^	0.042
TCMH	28.47 ± 0.02^b^	28.21 ± 0.02^c^	28.42 ± 0.11^c^	27.65 ± 0.02^a^	0.001
WBC	14 ± 0.58^b^	8.16 ± 0.34^c^	12.90 ± 0.96^c^	5.89 ± 0.24^a^	0.001
PN	48.29 ± 0.61^b^	52.43 ± 2.36^b^	41.57 ± 1.23^b^	48 ± 0.49^a^	0.001
PE	2.57 ± 0.20^a^	0.57 ± 0.20^c^	3 ± 0.22^c^	1 ± 0.38^ab^	0.001
L	49.14 ± 0.40^a^	45.71 ± 2.58^a^	54.57 ± 1.93^a^	50 ± 0.49^a^	0.001
M	0 ± 0	0.57 ± 0.30	1 ± 0.31	1 ± 0.38	0.085

Hb: Hemoglobin (g/dl); RBC: red blood cell (x10^6^/mm^3^); MCV: mean globular volume (fl); MCHC: mean corpuscular hemoglobin (g/dl); MHC: mean corpuscular hemoglobin concentration; WBC (x10^3^/L): white blood cell; PN: polymorphonuclear neutrophil (%); PE: polymorphonuclear eosinophil (%); L: lymphocytes (%); M: monocytes (%). Al: A. leiocarpus; Ad: A. digitata; NC: negative control; PC: positive control; ANOVA for independent samples. For the same row, values with the same superscript letter are not significantly different at *p* ≥ 0.05 (Duncan test).

## Data Availability

All data generated or analyzed during this study are included within the manuscript.
